# The Genetic Causes of Auditory Neuropathy: A Systematic Review

**DOI:** 10.3390/jcm15114260

**Published:** 2026-05-31

**Authors:** Nathania Yong, Michelle Cao, Erin Anderson, Lilian Downie, Gary Rance, Jinzi Bai, Karen Liddle, Antonia Howard, Libby Smith, Valerie Sung, Jing Wang

**Affiliations:** 1 Murdoch Children’s Research Institute, Parkville, VIC 3052, Australia; nathania.yong@mcri.edu.au (N.Y.); jing.wang@mcri.edu.au (J.W.); 2 The Royal Children’s Hospital, Parkville, VIC 3052, Australia; 3 Victorian Clinical Genetics Services, Parkville, VIC 3052, Australia; 4 Eugene Labs, Richmond, VIC 3121, Australia; 5 Department of Paediatrics, The University of Melbourne, Parkville, VIC 3052, Australia; 6 Department of Audiology and Speech Pathology, The University of Melbourne, Parkville, VIC 3010, Australia; 7 Child Health Research Centre, The University of Queensland, South Brisbane, QLD 4101, Australia; 8 Child Development Program, Queensland Children’s Hospital, Children’s Health Queensland, South Brisbane, QLD 4101, Australia

**Keywords:** auditory neuropathy, hearing loss, *OTOF*, genetics, diagnostic yield, next-generation sequencing

## Abstract

**Background/Objectives:** Auditory neuropathy is a form of hearing loss marked by preserved outer hair cell function and abnormal or absent auditory brainstem responses. Monogenic causes play a significant role in its aetiology. This systematic review aims to identify the genetic causes of auditory neuropathy reported in the literature and to determine the diagnostic yield of genetic testing in affected individuals. **Methods:** A systematic search of MEDLINE, Embase, and PubMed was conducted. Studies were included if participants had a diagnosis of auditory neuropathy and if genetic testing results were reported with variant interpretation based on American College of Medical Genetics and Genomics criteria. **Results/Discussion:** Twenty-nine studies involving 441 children and adults with auditory neuropathy were included. Overall, 21 different genes and 136 pathogenic and likely pathogenic variants were found to be causative of auditory neuropathy, with both syndromic and non-syndromic presentations. Variants in *OTOF* were the most common cause, responsible for 59% of all genetic diagnoses found. A genetic diagnosis was confirmed in 195 of 362 individuals who underwent genetic testing, resulting in a diagnostic yield of 54%. After adjusting for study bias and new gene associations with AN, the diagnostic yield was 31%. **Conclusions:** This review identifies gene and variant-level associations with auditory neuropathy that enhance our understanding of the condition. It highlights the high diagnostic yield of genetic testing in auditory neuropathy which supports consideration of genetic testing early in the diagnostic pathway. A genetic diagnosis may support precision-based approaches to treatment, including cochlear implants and participation in gene therapy trials.

## 1. Introduction

Auditory deprivation has a profound impact on many aspects of an individual’s life, impairing speech and language development, cognitive functioning, educational attainment, and leading to psychosocial consequences in children [1]. Auditory neuropathy (AN), a type of hearing loss, accounts for up to 10% of hearing loss cases [2,3,4,5,6]. First described in 1991 [7] and termed ‘auditory neuropathy’ in 1996 [8], the condition is characterised by preserved otoacoustic emissions (OAEs) and cochlear microphonics (CMs), alongside absent or severely distorted auditory brainstem responses (ABRs). This indicates a disruption in auditory neural activity from specific loss of cochlear inner hair cells (IHCs), a disruption at the synapse between IHCs and cochlear nerve fibres, at the spiral ganglion neurons, the auditory nerve fibres, or a combination of these sites [8].

AN has been associated with environmental and genetic causes and cochlear nerve deficiency [9]. Environmental risk factors include prematurity, hyperbilirubinaemia, and perinatal hypoxia [10,11,12]. Monogenic causes play a significant role in the aetiology of AN and may present as part of a complex phenotype, such as in the generalised neuropathic disorder of Charcot–Marie–Tooth syndrome, or as a non-syndromic hearing loss phenotype. Understanding the genetic basis of AN has significant clinical value and is of particular interest in the context of rapidly emerging gene therapies [13,14,15]. The identification of gene mutations responsible for auditory neuropathy has improved our understanding of the mechanism of disease which has led to advances in therapy. For example, gene therapy trials for otoferlin-mediated auditory neuropathy have shown promising results, paving the way for the development of gene therapies targeting other genetic causes of auditory neuropathy. Critical to further progress is a more comprehensive understanding of the genes and variants most commonly responsible for AN. Previous reviews in this area have not comprehensively collated these data, and additionally, they have primarily focused on gene-level data or on investigating single causative genes [12,16,17,18]. This review therefore seeks to provide a comprehensive and holistic overview of the genetic causes of AN that may guide directions for future research and aid genetic counselling and testing in affected individuals. It is important to note that there is a lack of data in the literature around the diagnostic yield of genetic testing in AN, the answer to which will inform the utility and place of genetic testing for AN.

Thus, this systematic review aims to identify all genetic causes of AN reported in the literature down to a variant level, and to determine the diagnostic yield of genetic testing in children and adults with AN.

## 2. Methods

This systematic review follows the Preferred Reporting Items for Systematic Reviews and Meta-Analyses (PRISMA) guidelines (Supplementary File S1). The protocol was registered in PROSPERO (registration number: CRD42024532206).

### 2.1. Search Strategy

A systematic search of MEDLINE (Ovid), Embase + Embase Classic (Ovid), and PubMed was conducted on 29 January 2024, without publication date restrictions. The search strategy combined thesaurus terms (MeSH and Emtree), keywords, and Boolean operators (Table S1). Primary search terms included ‘auditory diseases, central’, ‘vestibulocochlear nerve disease’, ‘perception deafness’ and ‘central hearing loss’. Keywords such as auditory neuropathy, auditory neuropathy spectrum disorder and auditory dyssynchrony were used. To refine results, genetic terms (e.g., gene, autosomal, syndrome, X-linked, mitochondrial, variant) were added. Filters excluded books, comments, editorials, guidelines, letters, preprints, and conference materials.

### 2.2. Eligibility Criteria

We included studies that reported genetic testing results of children or adults with stated AN or audiology findings consistent with AN (initially preserved OAEs or present CMs with absent or abnormal ABRs). To ensure reliability and clinical relevance, only studies that classified variants according to the American College of Medical Genetics and Genomics (ACMG) criteria—or intended to when no genetic diagnosis was made—were included [19]. Only English language studies were included. Literature reviews, narrative reviews, systematic reviews, guidelines, editorials, pre-prints, commentaries, conference abstracts, and letters were excluded.

### 2.3. Selection and Data Collection Process

Studies were uploaded to Covidence (Melbourne, Australia), which automatically removed duplicates. Two independent reviewers (NY, EA) screened titles and abstracts, with conflicts resolved by a third reviewer (MC, AH). Full texts were screened twice by two independent reviewers (NY, EA, MC or AH), with disagreements resolved through discussion.

All authors collaboratively designed a data extraction form. For each eligible study, one reviewer (NY, MC, EA, or JB) extracted data, which was cross-checked by another. Discrepancies were resolved through discussion. Data collected included the following:

- Report details—e.g., study design, country;
- Study characteristics—e.g., sample size, recruitment source;
- Hearing loss risk factors—e.g., birthweight, prematurity, hyperbilirubinemia;
- Audiology—e.g., OAEs, ABRs, CMs, cochlear nerve hypoplasia;
- Genetics—e.g., testing method, implicated gene/s, variants (Human Genome Variation Society nomenclature), ACMG classification, reference transcript.

### 2.4. Study Quality Assessment

Two reviewers (NY, EA, or JB) assessed methodological quality using the appropriate Joanna Briggs Institute critical appraisal tool [20]. Discrepancies were resolved via discussion. The tools evaluated aspects such as participant recruitment, validity of condition and outcome measurement, and appropriateness of statistical analysis. Items were rated ‘yes’, ‘no’, ‘unclear’, or ‘not applicable’. Items were removed if ‘not applicable’ across all relevant studies. Items regarding confounding were removed for cohort and cross-sectional studies as most did not have comparison groups, and assessment of confounding was considered less important when reporting genetic variants. Studies with ≥70% ‘yes’ ratings were considered higher quality.

### 2.5. Synthesis Methods

For each participant with AN and a genetic diagnosis, variant-level genetic data were extracted and tabulated. ‘Genetic diagnosis’ was defined as a case with variants consistent with the mode of inheritance of the gene and classified as pathogenic or likely pathogenic by ACMG criteria. Variants of uncertain significance (VUS) and unclassified variants were categorised as a diagnosis when found in the presence of a pathogenic or likely pathogenic variant in a recessive gene.

Genetic findings were grouped by phenotype: syndromic or non-syndromic AN. When phenotypic information was not reported, Online Mendelian Inheritance in Man (OMIM) was consulted to determine if the gene was associated with syndromic or non-syndromic hearing loss. If variants in the gene can cause syndromic and non-syndromic hearing loss for a given inheritance pattern, the case was categorised as non-syndromic for consistency.

## 3. Results

### 3.1. Study Selection

**Figure 1:**
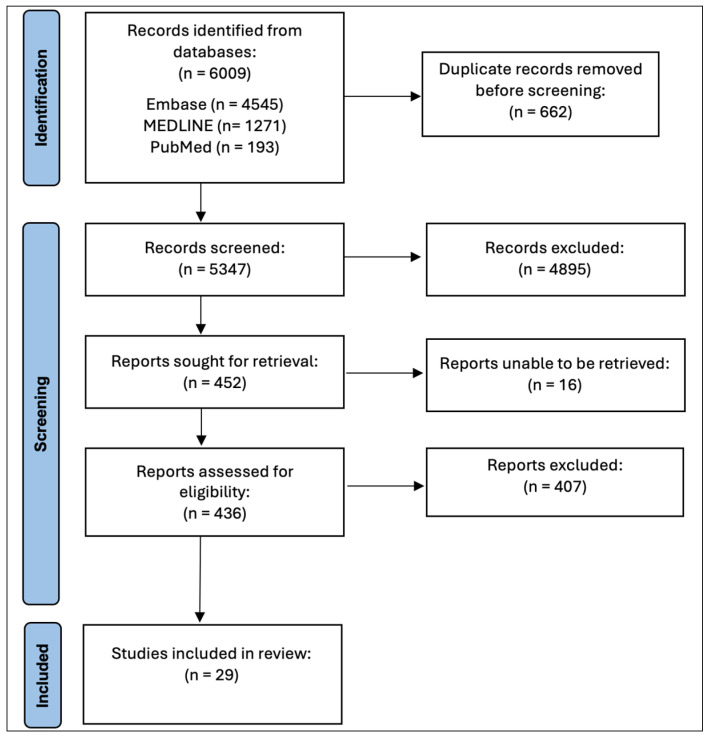
PRISMA flow diagram—adapted from Page et al. [21].

The PRISMA guidelines were consulted to conduct the review and systematically identify relevant articles [21]. A MEDLINE, Embase, and PubMed search resulted in 6009 records (Figure 1). Duplicates were removed, leaving 5347 articles. Title and abstract screening and initial full-text review resulted in 147 articles. After this initial full-text review, we excluded studies that did not use or plan to use ACMG criteria. Twenty-nine records underwent data extraction.

### 3.2. Study Characteristics

The study characteristics of included articles are summarised in Table 1. A total of 441 participants had AN. Most studies were case reports or case series (19/29). Studies were conducted across ten different countries, largely in Northeast Asia. The majority of articles used next-generation sequencing (NGS) methodologies, particularly exome sequencing or NGS-based panel testing.

### 3.3. Quality Assessment in Studies

The case reports were all assessed as higher quality with adequate clinical and diagnostic detail (Table S2). The quality of case series varied; five of thirteen were considered higher quality (Table S3). Most lacked complete or consecutive inclusion, often due to recruitment of a small number of distinct families. The single case–control study was not deemed higher quality (Table S4). Four of five cohort studies and all cross-sectional studies were considered higher quality (Tables S5 and S6). No studies were excluded from the review based on quality assessment, as all had thorough reporting of genetic findings using ACMG criteria.

### 3.4. Genetic Causes of Auditory Neuropathy

**Figure 2:**
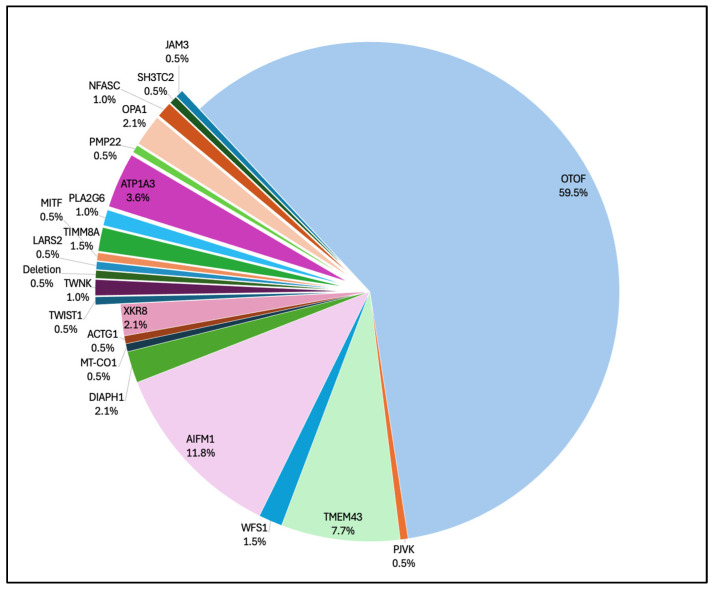
Proportion of auditory neuropathy cases attributed to each causative gene. Exploded slices represent syndromic genes.

One hundred ninety-five participants with AN received a genetic diagnosis, involving 21 genes and 136 different pathogenic and likely pathogenic variants (Table 2 and Table 3). Most cases, 64% (125/195), involved autosomal recessive inheritance across eight recessive genes. VUS *in trans* with a pathogenic or likely pathogenic variant were found in three cases [37,38,39], and unclassified variants found in combination with a likely pathogenic or pathogenic variant were reported in five cases [25,26,28,35,49]. *OTOF* was the most prevalent gene with 59% (116/195) of diagnoses attributed to it (Figure 2).

Variants in *OTOF* were the most common cause of non-syndromic AN, accounting for 69% (116/168) of non-syndromic AN diagnoses (Table 2). *AIFM1* was the second most common cause, accounting for 12% of all diagnoses (23/195) and 14% (23/168) of non-syndromic diagnoses.

Syndromic hearing loss was identified or suspected in 14% (27/195) of cases, based on reported phenotype or the gene involved. *ATP1A3* was the most common syndromic gene identified in association with AN, seen in 26% (7/27) of syndromic AN cases (Table 3). Symptoms consistent with *ATP1A3*-associated neurological disorder were reported in three of seven families with mutations in *ATP1A3* [34,43,45]. The second most common syndromic gene identified in association with AN was *OPA1*, found in 15% (4/27) of non-syndromic cases.

### 3.5. Diagnostic Yield of Genetic Testing in Auditory Neuropathy

Among 362 participants with AN who underwent genetic testing, 195 received a genetic diagnosis resulting in a combined diagnostic yield of 54% (195/362). If case reports and case series are excluded, the diagnostic yield decreases to 44% (111/251). These study types were excluded because their study samples are inherently biased. They selectively report AN patients with a confirmed genetic diagnosis or recruit from families that are likely to have a genetic diagnosis. Additionally, two larger studies recruited from populations biased towards a genetic diagnosis: Iwasa et al. [30] only recruited patients with two or more *OTOF* mutations, and Zanin et al. [46] recruited subjects already diagnosed with Auditory Neuropathy X-Linked 1. Removing these from the calculation lowers the yield to 32% (65/205). If not already excluded from the calculation, cases with variants in genes reported for the first time in association with AN were removed [45], resulting in a final diagnostic yield of 31% (64/204).

### 3.6. Ethnicity and Genotype

Among the papers reporting ethnicity, most cases were of Northeast Asian origin (Table 4). *OTOF* mutations were found in Chinese (number of cases = 44), Japanese (cases = 35), Taiwanese (cases = 18), and Korean (cases = 11) individuals. Only a small number of cases were from other regions.

### 3.7. Hearing Loss Characteristics and Genotype

Table 5 highlights features of hearing loss in the AN cases with a genetic diagnosis. In 24% of studies, ABR, CM, and/or OAE results were not reported for all participants; thus, the case was included in this review if the authors stated that they had AN [4,24,28,32,35,37,40]. The other cases had initially present OAEs or present CMs, and absent or abnormal ABRs. A common finding was disproportionately poorer speech discrimination relative to PTA/behavioural thresholds, particularly in noisy environments [26,31,41,43,49]. All AN cases with a genetic diagnosis presented with bilateral hearing loss, or lacked laterality data. Most *OTOF* cases had severe to profound, pre-lingual hearing loss. Nine temperature-sensitive AN cases, all *OTOF*-associated, showed fluctuating hearing loss, worsening during febrile episodes and improving upon temperature normalisation [26,48,49]. Post-lingual onset of hearing loss was described in more than two cases for the following genes: *XKR8*, *TMEM43*, *ATP1A3*, *AIFM1*, and *DIAPH1* [23,31,34,42,43,44,45,46]. Bilateral cochlear nerve hypoplasia (CNH) was reported in association with *OPA1*, *ATP1A3*, and *AIFM1* [29,42,43]. Wang et al. [42] reported bilateral CNH in three *AIFM1* cases, but it is unclear which of these cases had ACMG-classified variants and, therefore, a genetic diagnosis. Regardless, CNH amongst *AIFM1*-associated AN is consistent with the study by Zanin et al. [46], which found reduced apparent fibre density and therefore reduced neural fibre populations of the cochlear nerve in *AIFM1*-associated AN.

## 4. Discussion

This systematic review provides insight into the genotypic spectrum and diagnostic yield of testing in AN. Twenty-one genes and 136 different pathogenic or likely pathogenic variants are thought to be causative in 195 of 362 (54%) cases with AN. The number of genes found in this review is greater than previous reviews, which report 8–18 different causative genes or loci for AN [12,16,17,50]. This expansion may reflect greater utilisation of next-generation sequencing technologies, which have enabled more comprehensive sequencing of the genome. It may also be a reflection of the systematic methodology used in this review compared to previous reviews. The large majority of AN patients presented with a non-syndromic phenotype (86%) and autosomal recessive inheritance (64%)—consistent with a previous review [16]. *OTOF* was the most frequent causative gene, accounting for 59% of diagnoses in our cohort. *OTOF* mutations have been shown to impair synaptic exocytosis and neurotransmitter release, disrupting signal transmission to auditory nerve fibres—findings consistent with an auditory neuropathy [51]. Previous reviews recognise the prominence of *OTOF* as one of the first identified genetic causes of AN and a prominent contributor, but they do not provide quantitative estimates of its frequency in AN [12,50,52].

### 4.1. VUS and Unclassified Variants

VUS were identified in three cases [37,38,39]. In one case, reported by Liu et al. [37], a VUS was identified in *MYO3A* alongside two *OTOF* variants (one likely pathogenic and one VUS confirmed *in trans*). This suggests the biallelic *OTOF* variants were more likely the cause of this patient’s AN. For the other two cases, the remaining VUS were found *in trans* with pathogenic/likely pathogenic variants [38,39].

Unclassified variants were also found in the presence of pathogenic/likely pathogenic variants. Hosoya et al. [28] identified a likely pathogenic *OTOF* variant (c.3256G>A) and an unclassified variant (c.5816G>A), though no segregation analysis was carried out. Although the latter variant was unclassified by Hosoya et al. [28], it was classified as pathogenic in two other studies included in this review [30,33]. Zhu et al. [49] also reported an unclassified *OTOF* c.5098G>C variant *in trans* with the pathogenic c.4882C>A variant in four siblings. The unclassified variant was previously classified as likely pathogenic by Qiu et al. [38]. Li et al. [35] reported two siblings with infantile neuroaxonal dystrophy carrying a likely pathogenic *PLA2G6* variant (c.2249G>A) and an unclassified variant (c.196C>T) with no segregation in their analysis. The authors considered the unclassified variant ‘pathogenic’, despite no formal ACMG classification, as it was truncating.

### 4.2. Syndromic Genetic Causes

*ATP1A3* is associated with AN and was the most common gene associated with syndromic AN. It generally presents with the more complex phenotype of *ATP1A3*-associated neurological disorder, but this is variable, as some AN cases with *ATP1A3* variants denied neurological symptoms [34,43,45]. *OPA1* was the second most common syndromic gene identified, and the pathophysiological mechanisms by which *OPA1* causes AN have been well described [53,54,55]. The genes *JAM3*, *NFASC*, *PLA2G6*, and *TWIST1* were reported for the first time in association with AN [22,27,35,45].

Several genomic deletions were identified, including a large pathogenic deletion (chr7:4721914-5800744del) [45]. Of the genes within this CNV, *ACTB* was the most likely contributor due to its association with Baraitser–Winter cerebrofrontofacial syndrome, linked to SNHL, and has been previously reported as the responsible haploinsufficient gene in 7p22.1 microdeletion disorders [45,56].

One case had a dual diagnosis of Charcot–Marie–Tooth, caused by homozygous mutations in *SH3TC*, of which AN is a part, and an unrelated diagnosis of Ichthyosis Vulgaris associated with *FLG*—consistent with the reported phenotype [24].

### 4.3. Ethnic Predominance

The results reveal a predominance of Northeast Asian individuals (Table 4). All 18 Taiwanese patients with *OTOF* mutations identified by Lin et al. [36] were found to have the variant c.5098G>C. This is consistent with existing literature that shows that *OTOF* is a common deafness-associated gene in Taiwan, with evidence of a founder effect of the variant c.5098G>C in the population [57,58]. *AIFM1*, an X-linked gene, was also notably reported in Chinese cohorts. *AIFM1* has been identified as the gene responsible for Auditory Neuropathy X-linked 1 [59], which has only been reported in Chinese individuals so far [46]. This Northeast Asian predominance, however, may reflect national differences in research interest and capacity rather than a true increased prevalence in Northeast Asia.

### 4.4. Strengths and Limitations

This is the first systematic review to collate the genetic causes of auditory neuropathy reported in the literature. Novel contributions of this systematic review include the documentation of variants found in AN and the calculation of diagnostic yield. While previous reviews have identified genes associated with AN, this review also systematically compiles causative variants through analysis of classification and consistency with the gene’s inheritance pattern. Restricting inclusion to studies that utilised the ACMG framework allowed identification of variants more likely to be causative of AN. Although *OTOF* mutations have been the focus of many studies, this review identifies additional genetic causes. Lastly, this review aimed to consolidate the existing literature into a comprehensive, accessible resource to help guide decisions on when and if genetic testing is offered and the interpretation of results.

Several limitations must be considered. Firstly, studies testing a cohort of individuals with hearing loss, including some with AN, would have been screened out if AN was not mentioned in the title or abstract. Secondly, 24% of studies did not report ABR, CM, and/or OAE results for all participants. Consequently, independent verification of the presence of AN was not possible in these cases. Thirdly, heterogeneity in genetic testing methods restricts the comparability of studies and interpretation of a combined diagnostic yield. Fourthly, the literature had a predominance of case reports and series and predominantly recruited individuals from Northeast Asia. This may indicate reporting and selection bias in the literature and may limit the generalisability of findings. Fifthly, many included studies, particularly case reports and case series, involved selective populations biased towards identifying a genetic cause. For example, Jang et al. [31] contributed fourteen AN cases from two families, all with the same genotype. Such overrepresentation of positive findings may have inflated the overall diagnostic yield. When case reports and case series were excluded, the diagnostic yield decreased to 44% (111/251), and further to 32% (65/205) when two larger studies with populations biased towards a genetic diagnosis were removed [30,46]. Removing cases where a gene was reported to be associated with AN for the first time lowered the yield to 31% (64/204) [45]. Notwithstanding these limitations, the diagnostic yield is still high, supporting the value of genetic testing in identifying individuals who may benefit from targeted interventions.

### 4.5. Implications and Future Directions

This systematic review provides a valuable resource for aiding genetic testing and counselling of AN patients. The observed diagnostic yield of 54%, or even the adjusted yield of 31%, demonstrates the utility of genetic testing in AN. Earlier and more widespread genetic testing could enable earlier hearing interventions. An early genetic diagnosis can also streamline the process by reducing unnecessary testing and associated healthcare costs [60]. It also has personal utility in providing reassurance to families through diagnostic certainty and empowering them to make informed reproductive choices in the future [61].

In precision medicine, understanding genetic causes can help predict the value of different interventions, particularly in the assessment of candidacy for cochlear implantation (CI) as outcomes vary by aetiology, likely reflecting different lesion sites [34,36,55,62,63]. Genetic and audiological diagnostics help localise the site of the lesion and therefore may help predict CI outcomes [12,64]. For example, *OTOF* mutations affect the IHC ribbon synapse [51], and such synaptic lesions typically have good CI outcomes, as electrical stimulation up to the level of the spiral ganglion neurons is expected to bypass the lesion [12,65]. By contrast, lesions affecting the auditory nerve/brainstem may show poor CI performance as the CI-generated signal still needs to pass through a disordered system [65]. Table S7 provides further examples of the likely lesion site for genes identified in this review.

Gene therapy is emerging as a promising treatment for AN. An improved understanding of genetic etiology may help guide the development of these targeted gene therapies [12]. Gene therapies are in preclinical and early clinical stages. Small trials of gene therapy for *OTOF*-associated AN have demonstrated encouraging preliminary findings including improvements in hearing, speech, and sound localisation [13,14]. Therapy is now available, as evidenced by the U.S Food and Drug Administration recently approving Otarmeni as hearing loss gene therapy for those with molecularly confirmed biallelic variants in OTOF [66]. In this context, early genetic testing will help eligible patients access new therapies. The high diagnostic yield identified in this review suggests the clinical value of genetic testing in AN for establishing a genetic diagnosis, potentially identifying patients who may be suitable candidates for future gene therapy trials as these therapies continue to be developed. Additionally, genotype correlations identified here may help inform future gene therapy research.

Future research should prioritise recruitment of larger, demographically representative cohorts to validate genotype–phenotype correlations and provide a more representative calculation of diagnostic yield. Standardised methods—including consistent application of ACMG criteria and clear audiological evidence of AN—are important for meaningful between-study comparison. Further, future studies that include detailed auditory–genotypic associations would be important. Emerging gene–disease associations (e.g., *JAM3*, *NFASC*, *PLA2G6*, *TWIST1*) also warrant further investigation. Additionally, functional studies investigating the mechanism of specific gene mutations will help clarify lesion sites and inform therapeutic options.

This systematic review confirms the genetic heterogeneity of AN, and the high diagnostic yield emphasises the utility of genetic testing in this condition. Efforts to advance our understanding of the genetic basis of AN supports the development of more advanced therapeutic strategies in the future, such as gene therapy.

## Figures and Tables

**Table 1:** Study characteristics of the included articles.

Author (Year)	Study Design	Location of Study	Ethnicity of AN Participants with a Genetic Diagnosis	Source Population of Participants	No. of Participants with AN	Genetic Testing Method	No. of Participants with AN Who Had Genetic Testing
Abdallah Moady (2023) [22]	Case report	Israel	North African and Middle Eastern	Not reported	1 Low birthweight *	- Sanger sequencing (JAM3) - NGS-based panel (300 hearing loss genes)	1
Batissoco (2022) [4]	Cross-sectional	Brazil	South American	HL subjects referred to the Genetic Deafness Counselling Unit	19	- Exome sequencing (1 case) - Sanger sequencing *OTOF* (1 case)	2
Chen (2023) [23]	Case series	China	Northeast Asian (Chinese)	Participants presenting to an outpatient clinic for genetic counselling	4 (1 family)	- Exome sequencing	4
Chhajed (2022) [24]	Case report	India	Not reported	Participant presented to a paediatric outpatient department with motor delay and floppiness of lower limbs since birth	1	- Exome sequencing	1
Domínguez-Ruiz (2022) [25]	Two distinct cohorts and a case-series component	Spain Denmark Italy	Southern and Eastern European (Italian)	Isolated AN simplex cases in whom *OTOF* variants had been excluded	84	- Sanger sequencing (*PJVK*)	84
Forli (2023) [26]	Case report	Italy	Southern and Eastern European	Participant underwent audiological evaluation for hearing difficulties at Otolaryngology, Audiology, and Phoniatrics department, Pisa University Hospital	1	- Targeted NGS panel (37 deafness-associated genes) - CNV analysis	1
Harper (2020) [27]	Case report	United States	Not reported	Not reported	2 (1 family) Low birthweight Prematurity Neonatal ICU admission *	- Exome sequencing	2
Hosoya (2018) [28]	Case series	Japan	Not reported	CI recipients (December 2008–November 2016) with MED-EL or Advanced Bionics implants. All diagnosed with *OTOF*, *GJB2*, or *SLC26A4* mutations, or maternal CMV infection.	4	- Sanger sequencing	4
Ideura (2019) [29]	Cross-sectional	Japan	Northeast Asian (Japanese)	Probands with possible syndromic hearing loss from 67 research institutes in Japan	1	- Targeted NGS panel (36 syndromic hearing loss associated genes) - CNV analysis	1
Iwasa (2022) [30]	Cohort study (retrospective)	Japan	Northeast Asian (Japanese)	Patients with ≥2 *OTOF* mutations registered in a database February 2012–December 2020 from 96 otolaryngology departments in Japan	35	- Targeted NGS panel (68 genes reported to cause non-syndromic HL) - Sanger sequencing of variants identified by NGS panel	35
Jang (2021) [31]	Case series	China South Korea	Northeast Asian (Chinese, Korean)	Not reported	14 (2 families)	- Exome sequencing - Sanger sequencing of variants identified by exome sequencing	14
Jiang (2022) [32]	Case report	China	Northeast Asian (Chinese)	Not reported	3 (1 family)	- Exome sequencing	3
Kim (2018) [33]	Case series	South Korea	Northeast Asian (Korean)	Families with hereditary HL (sporadic or AR) from Seoul National University Hospital and Seoul National University Bundang Hospital (June 2015–March 2017).	11	- Screening panel (11 variants in 5 deafness genes) or Sanger sequencing of p.Arg1939Gln of *OTOF* - If a potential candidate variant of *OTOF* was detected, then *OTOF* sanger sequencing was performed to find another candidate variant - If no variant was detected in screening, targeted or whole exome sequencing followed	11
Kim (2023) [34]	Cohort study (retrospective)	South Korea	Northeast Asian (Korean)	Database of probands with HL who under-went molecular genetic testing (March 2010–September 2022)	15	- Exome sequencing	15
Li (2020) [35]	Case report	Malaysia	Southeast Asian	Patients referred to a paediatric department for psychomotor regression and progressive central hypotonia	2 (1 family)	- Exome sequencing	2
Lin (2022) [36]	Cohort study (retrospective)	Taiwan	Northeast Asian (Taiwanese)	AN patients who had undergone CI at three referral centres from 2001–2021	36	- Targeted NGS panel (220 deafness genes)	36
Liu (2022) [37]	Cross-sectional	China	Northeast Asian (Chinese)	Prelingual, non-syndromic Han Chinese children with sensorineural hearing loss in special education schools, Zhejiang Province (March 2018–August 2018)	1	- Exome sequencing	1
Qiu (2019) [38]	Case series	China	Northeast Asian (Chinese)	Not reported	2	- Targeted NGS panel (162 deafness-related genes)	2
Sun (2022) [39]	Case series	China	Northeast Asian (Chinese)	Patients undergoing genetic counselling at the department of otolaryngology-head and neck surgery, Xin Hua Hospital	9	- Targeted NGS panel (140 deafness-causing genes for one case, 415 deafness-causing genes for the others)	9
Tang (2015) [40]	Case series	United States	Not reported	HL patients of suspected genetic aetiology from the Baylor College of Medicine and Texas Children’s Hospital clinical care centres	4	- Sequencing of *GJB2* - Sanger sequencing of coding and near-coding regions of additional genes based on the patient’s clinical findings	4
Wang (2018) [41]	Case series	China	Northeast Asian (Chinese)	Not reported	3 (1 family)	- Targeted NGS panel (all known genes associated with syndromic and non-syndromic hearing loss at hereditaryhearingloss.org)	3
Wang (2020) [42]	Cohort study	China	Northeast Asian (Chinese)	Patients with AIFM1 mutations and AN from Chinese PLA General Hospital (April 1997 to June 2019)	50 (36 families)	- Genome sequencing	20
Wang (2021) [43]	Case series	China	Northeast Asian (Chinese)	Patients with AN at the Institute of Otolaryngology, Chinese PLA General Hospital	4	- Genome sequencing - Targeted NGS panel	4
Wu (2020) [44]	Case series	China	Northeast Asian (Chinese)	A large Chinese family from a previous study	4 (1 family)	- Exome sequencing	4
Wu (2023) [45]	Cohort study	China	Northeast Asian (Chinese)	Patients diagnosed with AN and received cochlear implants at Chinese PLA General Hospital (August 2010–November 2020)	75	- Exome sequencing - CNV analysis	46
Zanin (2020) [46]	Case-control	China	Northeast Asian (Chinese)	Individuals with Auditory Neuropathy X-linked 1	11	- Exome sequencing	11
Zhai (2020) [47]	Case series	China	Northeast Asian (Chinese)	3 families with non-syndromic HL	4 (3 families)	- Exome sequencing	4
Zhang (2016) [48]	Case series (with genetic association analysis)	China	Northeast Asian (Chinese)	Patients with AN collected through a national network for collecting genetic resources for hearing loss from 2004–2013, maintained by China PLA Institute of Otolaryngology	37	- Sanger sequencing (*OTOF*)	34
Zhu (2021) [49]	Case series	China	Northeast Asian (Chinese)	Patients attended an outpatient clinic due to hearing issues when unwell	4 (1 family)	- Targeted NGS (159 deafness-related nuclear genes, 6 deafness-related mitochondrial regions, 3 miRNAs) - Exome sequencing (1 case)	4 (2 are monozygotic twins)

* Presence of risk factors amongst AN cases with a genetic diagnosis. Low birthweight (<2.5 kg), prematurity (<37 weeks gestation), hyperbilirubinaemia, neonatal ICU/special care nursery admission.

**Table 2:** Genotypes of the non-syndromic AN cases.

Author (Year)	No. of Cases	Gene	Transcript	Variant 1	Class ACMG	Variant 2	Class ACMG	Variant 3	Class ACMG	Segregation Analysis
Batissoco (2022) [4]	1	*OTOF*	NM_194248.3	c.3049G p.Glu1017Ter	P	c.3400C>T p.Arg1134Ter	P	N/A	N/A	No
	1	*OTOF*	NM_194248.3	c.2153G>A p.Trp718Ter	P	c.3332C>T p.Pro1111Leu	LP	N/A	N/A	No
Chen (2023) [23]	4	*XKR8*	NM_018053	c.710G>A p.Trp237Ter	LP	N/A	N/A	N/A	N/A	Done for 3/4
Domínguez-Ruiz (2022) [25]	1	*PJVK*	NM_001042702.3	c.880C>G p.His294Asp	LP	c.950del p.Phe317SerfsTer20	Not reported	N/A	N/A	Yes
Forli (2023) [26]	1	*OTOF*	NM_194248.2	c.2521G>A p.Glu841Lys	P	c.(897+1_898-1)_(1579+1_1580-1)del	Not reported	N/A	N/A	Incomplete
Hosoya (2018) [28]	1	*OTOF*	NM_194248.2	c.3256G>A p.Gly1086Arg	LP	c.5816G>A p.Arg1939Gln	Not reported	N/A	N/A	No
Iwasa (2022) [30]	15	*OTOF*	NM_001287489	c.5816G>A p.Arg1939Gln	P	Homozygous	N/A	N/A	N/A	No
	2	*OTOF*	NM_001287489	c.5816G>A p.Arg1939Gln	P	c.3192C>G p.Tyr1064Ter	P	N/A	N/A	No
	1	*OTOF*	NM_001287489	c.5816G>A p.Arg1939Gln	P	c.1465C>T p.Pro489Ser	LP	N/A	N/A	No
	1	*OTOF*	NM_001287489	c.5816G>A p.Arg1939Gln	P	c.5374C>T p.Arg1792Cys	LP	N/A	N/A	No
	1	*OTOF*	NM_001287489	c.5816G>A p.Arg1939Gln	P	c.4346_4347insGCAT p.Ile1449fs	P	N/A	N/A	No
	2	*OTOF*	NM_001287489	c.5816G>A p.Arg1939Gln	P	c.3214C>T p.Gln1072Ter	P	N/A	N/A	No
	2	*OTOF*	NM_001287489	c.5816G>A p.Arg1939Gln	P	c.5815C>T p.Arg1939Trp	LP	N/A	N/A	No
	2	*OTOF*	NM_001287489	c.5816G>A p.Arg1939Gln	P	c.1422T>A p.Tyr474Ter	P	N/A	N/A	No
	1	*OTOF*	NM_001287489	c.5816G>A p.Arg1939Gln	P	c.2690_2700del p.Arg897fs	P	N/A	N/A	No
	1	*OTOF*	NM_001287489	c.5816G>A p.Arg1939Gln	P	c.5566C>T p.Arg1856Trp	LP	N/A	N/A	No
	1	*OTOF*	NM_001287489	c.5816G>A p.Arg1939Gln	P	c.2269_2274delinsA p.Glu757fs	P	N/A	N/A	No
	1	*OTOF*	NM_001287489	c.5816G>A p.Arg1939Gln	P	c.5838G>A p.Trp1946Ter	P	N/A	N/A	No
	1	*OTOF*	NM_001287489	c.5816G>A p.Arg1939Gln	P	c.5500delG p.Asp1834fs	P	N/A	N/A	No
	1	*OTOF*	NM_001287489	c.5567G>A p.Arg1856Gln	P	c.1422T>A p.Tyr474Ter	P	N/A	N/A	No
	2	*OTOF*	NM_001287489	c.5815C>T p.Arg1939Trp	LP	c.5728G>A p.Glu1910Lys	LP	N/A	N/A	No
	1	*OTOF*	NM_001287489	c.4718T>C p.Ile1573Thr	LP	c.4129_4138del p.Ala1377fs	P	N/A	N/A	No
Jang (2021) [31]	14	*TMEM43*	NM_024334	c.1114C>T p.Arg372Ter	P	N/A	N/A	N/A	N/A	Yes
Jiang (2022) [32]	3	*OTOF*	NM_194248.2	c.3277G>A p.Glu1093Lys	LP	c.4024-4G>T	LP	c.898-2A>G	P	Yes
Kim (2018) [33]	2	*OTOF*	NM_001287489	c.5816G>A p.Arg1939Gln	P	Homozygous	N/A	N/A	N/A	No
	1	*OTOF*	NM_001287489	c.5816G>A p.Arg1939Gln	P	Chr2:26710657~26706557	P	N/A	N/A	No
	2	*OTOF*	NM_001287489	c.5816G>A p.Arg1939Gln	P	c.5566C>T p.Arg1856Trp	P	N/A	N/A	No
	1	*OTOF*	NM_001287489	c.5816G>A p.Arg1939Gln	P	c.2521G>A p.Glu841Lys	P	N/A	N/A	No
	1	*OTOF*	NM_001287489	c.5816G>A p.Arg1939Gln	P	c.3032T>C p.Leu1011Pro	P	N/A	N/A	Yes
	1	*OTOF*	NM_001287489	c.5791C>A p.Pro1931Thr	P	c.2521G>A p.Glu841Lys	P	N/A	N/A	Yes
	1	*OTOF*	NM_001287489	c.3192C>G p.Tyr1064Ter	P	Homozygous	N/A	N/A	N/A	No
	1	*OTOF*	NM_001287489	c.5534G>A p.Gly1845Glu	P	c.3032T>C p.Leu1011Pro	P	N/A	N/A	No
	1	*OTOF*	NM_001287489	c.2521G>A p.Glu841Lys	P	c.4227+5G>C	LP	N/A	N/A	No
Kim (2023) [34]	1	*TMEM43*	NM_024334.2	c.1114C>T p.Arg372Ter	P	N/A	N/A	N/A	N/A	No
Lin (2022) [36]	7	*OTOF*	Not reported	c.5098G>C	LP/P †	Homozygous	N/A	N/A	N/A	No
	1	*OTOF*	Not reported	c.1498C>T	LP/P †	c.5098G>C	LP/P †	N/A	N/A	No
	2	*OTOF*	Not reported	c.2521G>A	LP/P †	c.5098G>C	LP/P †	N/A	N/A	No
	2	*OTOF*	Not reported	c.3704_3719del	LP/P †	c.5098G>C	LP/P †	N/A	N/A	No
	1	*OTOF*	Not reported	c.3864G>A	LP/P †	c.5098G>C	LP/P †	N/A	N/A	No
	1	*OTOF*	Not reported	c.4030C>T	LP/P †	c.5098G>C	LP/P †	N/A	N/A	No
	1	*OTOF*	Not reported	c.4961-1G>A	LP/P †	c.5098G>C	LP/P †	N/A	N/A	No
	1	*OTOF*	Not reported	c.5000C>A	LP/P †	c.5098G>C	LP/P †	N/A	N/A	No
	1	*OTOF*	Not reported	c.5098G>C	LP/P †	c.5203C>T	LP/P †	N/A	N/A	No
	1	*OTOF*	Not reported	c.5098G>C	LP/P †	c.5566C>T	LP/P †	N/A	N/A	No
	1	*WFS1*	Not reported	c.2051C>T p.Ala684Val	P	N/A	N/A	N/A	N/A	No
Liu (2022) [37]	1	*OTOF*	NM_194248	c.4691G>A p.Trp1564Ter	P	c.3928_3930dup p.Lys1310dup	VUS/LP ‡	N/A	N/A	Yes
		*MYO3A*	NM_017433	c.610G>A p.Asp204Asn	VUS/LP ‡	N/A	N/A	N/A	N/A	Yes
Qiu (2019) [38]	1	*OTOF*	NM_194248	c.4748G>A p.Arg1583His	LP	c.2523+1G>T	LP	N/A	N/A	Yes
	1	*OTOF*	NM_194248	c.5098G>C p.Glu1700Gln	LP	c.5248G>C p.Asp1750His	VUS	N/A	N/A	Yes
Sun (2022) [39]	1	*OTOF*	NM_194248	c.5308C>T p.Gln1770Ter	LP	c.4236del p.Glu1414SerfsTer108	LP	N/A	N/A	Yes
	1	*OTOF*	NM_194248	c.4225A>T p.Lys1409Ter	P	c.2406+2_2406+3insT	LP	N/A	N/A	Yes
	1	*OTOF*	NM_194248	c.4961-3C>G	LP	c.4091-1G>A	LP	N/A	N/A	Yes
	1	*WFS1*	NM_006005	c.937C>T p.His313Tyr	LP	N/A	N/A	N/A	N/A	Yes
	1	*WFS1*	NM_006005	c.2029G>A p.Ala677Thr	LP	N/A	N/A	N/A	N/A	Yes
Tang (2015) [40]	1	*OTOF*	NM_194248.1	c.897+1G>T	P	c.2485C>T p.Gln829Ter	P	N/A	N/A	No
Wang (2018) [41]	2	*OTOF*	NM_001287489	c.1550T>C p.Leu517Pro	P	c.5900-5902delTCA p.Ile1967del	P	N/A	N/A	Yes
	1	*OTOF*	NM_001287489	c.1550T>C p.Leu517Pro	P	Homozygous	N/A	N/A	N/A	Yes
Wang (2020) [42]	1	*AIFM1*	Not reported	c.547A>T p.Thr183Ser	P	N/A	N/A	N/A	N/A	Yes
	1	*AIFM1*	Not reported	c.881G>A p.Arg294Gln	P	N/A	N/A	N/A	N/A	Yes
	1	*AIFM1*	Not reported	c.890A>T p.Lys297Ile	P	N/A	N/A	N/A	N/A	Yes
	2	*AIFM1*	Not reported	c.912C>G p.Ile304Met	P	N/A	N/A	N/A	N/A	Yes
	1	*AIFM1*	Not reported	c.997C>T p.Leu333Phe	P	N/A	N/A	N/A	N/A	Yes
	1	*AIFM1*	Not reported	c.1394C>T p.Ala465Val	P	N/A	N/A	N/A	N/A	Yes
	2	*AIFM1*	Not reported	c.1678T>C p.Tyr560His	P	N/A	N/A	N/A	N/A	Yes
Wu (2020) [44]	4	*DIAPH1*	NM_005219.4	c.3551_3552del p.Glu1184AlafsTer11	P	N/A	N/A	N/A	N/A	Yes
Wu (2023) [45]	1	*OTOF*	NM_194248.2	c.4493T>A p.Val1498Glu	LP	c.5782C>T p.Arg1928Cys	LP	N/A	N/A	Yes
	1	*OTOF*	NM_194248.2	c.5098G>C p.Glu1700Gln	LP	c.2407-2delA	P	N/A	N/A	Yes
	1	*OTOF*	NM_194248.2	c.5570G>A p.Gly1857Asp	LP	c.5212_5214delATC p.Ile1738del	LP	N/A	N/A	Yes
	1	*OTOF*	NM_194248.2	c.3399C>A p.Tyr1133Ter	P	c.5833del p.Ile1945SerfsTer4	P	N/A	N/A	Yes
	1	*OTOF*	NM_194248.2	c.3674C>G p.Ser1225Cys	LP	c.3592dup p.Leu1198ProfsTer94	P	N/A	N/A	Yes
	1	*OTOF*	NM_194248.2	c.5566C>T p.Arg1856Trp	LP	c.764A>C p.Gln255Pro	LP	N/A	N/A	Yes
	1	*OTOF*	NM_194248.2	c.4030C>T p.Arg1344Ter	P	c.1432T>C p.Trp478Arg	LP	N/A	N/A	Yes
	1	*OTOF*	NM_194248.2	c.4110_4120dup p.Lys1374ArgfsTer152	P	c.2215-1G>C	P	N/A	N/A	Yes
	2	*OTOF*	NM_194248.2	c.5815C>T p.Arg1939Trp	P	Homozygous	N/A	N/A	N/A	Yes
	1	*OTOF*	NM_194248.2	c.5291+1G>T	P	c.5566C>T p.Arg1856Trp	LP	N/A	N/A	Yes
	1	*OTOF*	NM_194248.2	c.5203C>T p.Arg1735Trp	LP	c.2985C>A p.Cys995Ter	P	N/A	N/A	Yes
	1	*OTOF*	NM_194248.2	c.5108_5114delinsTCTTCCTGGG,p.Arg1703_Glu1705delinsLeuPheLeuGly	LP	c.709C>T p.Arg237Ter	P	N/A	N/A	Yes
	1	*MT-CO1*	Not reported	m.A7445G	P	N/A	N/A	N/A	N/A	Yes
	1	*AIFM1*	NM_004208.3	c.434C>T p.Ala145Val	LP	N/A	N/A	N/A	N/A	Yes
	1	*AIFM1*	NM_004208.3	c.1773C>G p.Ile591Met	LP	N/A	N/A	N/A	N/A	Yes
	1	*AIFM1*	NM_004208.3	c.649A>G p.Arg217Gly	LP	N/A	N/A	N/A	N/A	Yes
	1	*ACTG1*	NM_001199954.1	c.377C>T p.Thr126Ile	LP	N/A	N/A	N/A	N/A	Yes—*de novo*
Zanin (2020) [46]	1	*AIFM1*	Not reported	c.1394C>T p.Ala465Val	P	N/A	N/A	N/A	N/A	No
	1	*AIFM1*	Not reported	c.1678T>C p.Tyr560His	P	N/A	N/A	N/A	N/A	No
	2	*AIFM1*	Not reported	c.1264C>T p.Arg422Trp	P	N/A	N/A	N/A	N/A	No
	1	*AIFM1*	Not reported	c.1773C>G p.Ile591Met	P	N/A	N/A	N/A	N/A	No
	1	*AIFM1*	Not reported	c.1492G>A p.Val498Met	P	N/A	N/A	N/A	N/A	No
	2	*AIFM1*	Not reported	c.1030C>T p.Leu344Phe	P	N/A	N/A	N/A	N/A	No
	3	*AIFM1*	Not reported	c.1265G>A p.Arg422Gln	P	N/A	N/A	N/A	N/A	No
Zhai (2020) [47]	2	*OTOF*	NM_194248.3	c.2688del p.Lys896AsnfsTer104	P	Homozygous	N/A	N/A	N/A	Yes
	1	*OTOF*	NM_194248.3	c.4960G>A p.Gly1654Ser	P	c.1469C>G p.Pro490Arg	LP	N/A	N/A	Yes
	1	*OTOF*	NM_194248.3	c.2675A>G p.Lys892Arg	LP	c.2977_2978del p.Gln994ValfsTer7	P	N/A	N/A	Yes
Zhang (2016) [48]	1	*OTOF*	NM_194248.1	c.2901C>G p.Tyr967Ter	P	c.5666G>C p.Trp1889Ser	P	N/A	N/A	Yes
	1	*OTOF*	NM_194248.1	c.1539_1554del15 p.His513del	P	c.5330A>G p.Asp1777Gly	P	N/A	N/A	Yes
	1	*OTOF*	NM_194248.1	c.3570+2T>C	P	c.4225A>T p.Lys1409Ter	P	c.4981G>A p.Glu1661Lys	P	Yes
	1	*OTOF*	NM_194248.1	c.4023+1G>A	P	Homozygous	N/A	N/A	N/A	Yes
	1	*OTOF*	NM_194248.1	c.3399C>A p.Tyr1133Ter	P	c.5833delG p.Val1945Serfs	P	N/A	N/A	Yes
	1	*OTOF*	NM_194248.1	c.3316_3321insC p.Ile1108HisfsTer69	P	c.4023+1G>A	P	N/A	N/A	Yes
	1	*OTOF*	NM_194248.1	c.4493T>A p.Val1498Glu	P	c.5782C>T p.Arg1928Cys	P	N/A	N/A	Yes
	1	*OTOF*	NM_194248.1	c.4033C>T p.Gln1345Ter	P	c.5197G>A p.Glu1733Lys	P	N/A	N/A	Yes
	1	*OTOF*	NM_194248.1	c.765+1G>C	P	c.2377G>T p.Glu793Ter	P	N/A	N/A	Yes
	1	*OTOF*	NM_194248.1	c.2215-1G>C	P	c.4747C>T p.Arg1583Cys	P	N/A	N/A	Yes
	1	*OTOF*	NM_194248.1	c.2975_2978delAG p.Gln994ValfsTer6	P	c.4819C>T p.Arg1607Trp	P	N/A	N/A	Yes
	1	*OTOF*	NM_194248.1	c.4819C>T p.Arg1607Trp	P	Homozygous	N/A	N/A	N/A	Yes
	1	*OTOF*	NM_194248.1	c.2093G>C p.Arg698Thr	P	c.4981G>A p.Glu1661Lys	P	N/A	N/A	Yes
	1	*OTOF*	NM_194248.1	c.2382_2383delC p.Leu795SerfsTer5	P	c.1621G>A p.Gly541Ser	P	N/A	N/A	Yes–c.1621G>A *de novo*
Zhu (2021) [49]	4	*OTOF*	NM_194248.2	c.4882C>A p.Pro1628Thr	P	c.5098G>C p.Glu1700Gln	Not reported	N/A	N/A	Yes

P: pathogenic; LP: likely pathogenic; VUS: variant of uncertain significance; N/A: not applicable. †: Lin et al. [36] reported their variants as likely pathogenic or pathogenic (LP/P) by ACMG criteria, though it is unclear which variants are LP and which are P. ‡: The authors of this review assume the classification VUS/LP by Liu et al. [37] represents a borderline classification; however, for the purposes of data synthesis, this review will consider these variants as VUS.

**Table 3:** Genotypes of the syndromic AN cases.

Author (Year)	No. of Cases	Syndrome	Gene	Transcript	Variant 1	Class ACMG	Variant 2	Class ACMG	Segregation
Abdallah Moady (2023) [22]	1	Haemorrhagic destruction of the brain, subependymal calcification, and cataracts OMIM:606871	*JAM3*	NM_032801	c.745dup p.Val249GlyfsTer28	P	Homozygous	N/A	Yes
Chhajed (2022) [24]	1	Charcot–Marie–Tooth disease Type 4C OMIM:601596 Ichthyosis Vulgaris OMIM:135940	*SH3TC2*	NM_024577.3	c.3325C>T p.Arg1109Ter	P	Homozygous	N/A	Incomplete
			*FLG*	NM_002016.1	c.3325C>T p.Arg1109Ter	P	N/A	N/A	
Harper (2020) [27]	2	Neurodevelopmental Disorder with Central and Peripheral Motor Dysfunction OMIM:609145	*NFASC*	NM_015090.3	c.2771delC p.Pro924ArgfsTer35	P	Homozygous	N/A	Yes
Ideura (2019) [29]	1	Optic atrophy plus syndrome OMIM:605290	*OPA1*	NM_015560	c.892A>C p.Ser298Arg	LP	N/A	N/A	No
Kim (2023) [34]	1	Optic atrophy plus syndrome OMIM:605290	*OPA1*	NM_015560.3	c.892A>C p.Ser298Arg	LP	N/A	N/A	No
	1	Optic atrophy plus syndrome OMIM:605290	*OPA1*	NM_015560.3	c.1334G>A p.Arg445His	P	N/A	N/A	No
	1	Charcot–Marie–Tooth disease Type 1A OMIM:601097	*PMP22*	Not reported	Chr17:(14140179_14204367)_(15472344_15487200)deletion	P	N/A	N/A	No
	2	ATP1A3-associated neurological disorder MONDO:0700002	*ATP1A3*	NM_152296.5	c.2452G>A p.Glu818Lys	P	N/A	N/A	No
Li (2020) [35]	2	Infantile neuroaxonal dystrophy OMIM:256600	*PLA2G6*	Not reported	c.2249G>A p.Cys750Tyr	LP	c.196C>T p.Gln66X	Not reported	No
Lin (2022) [36]	1	Optic atrophy plus syndrome OMIM:605290	*OPA1*	Not reported	c.1414T>C p.Cys472Arg	P	N/A	N/A	No
Sun (2022) [39]	1	Mohr–Tranebjaerg syndrome OMIM:300356	*TIMM8A*	NM_004085	c.61_62insGGACCCGCAGTTGCAGC, p.His21ArgfsTer11	LP	N/A	N/A	Yes—*de novo*
	1	Waardenburg syndrome 2A OMIM:156845	*MITF*	NM_000248	c.733delA p.Thr245ProfsTer3	LP	N/A	N/A	Yes
	1	OMIM:604544	*LARS2*	NM_015340	c.1987C>T p.Arg663Trp	LP	c.764C>T, p.Ala255Val	VUS	Yes
Wang (2021) [43]	4	ATP1A3-associated neurological disorder MONDO:0700002	*ATP1A3*	NM_152296.4	c.2452G>A p.Glu818Lys	P	N/A	N/A	Yes—*de novo*
Wu (2023) [45]	1	Mohr–Tranebjaerg syndrome OMIM:300356	*TIMM8A*	NM_004085.3	c.133-2A>G	P	N/A	N/A	Yes
	1	Mohr–Tranebjaerg syndrome OMIM:300356	*TIMM8A*	NM_004085.3	c.223C>T p.Gln75Ter	P	N/A	N/A	Yes
	1	ATP1A3-associated neurological disorder MONDO:0700002	ATP1A3	NM_152296.4	c.2452G>A p.Glu818Lys	P	N/A	N/A	Yes
	1		chr7:4721914-5800744del			P	N/A	N/A	Yes—*de novo*
	1	Saethre–Chotzen syndrome OMIM:601622	*TWIST1*	NM_000474.3	c.309C>A p.Tyr103Ter	P	N/A	N/A	No
	1	Perrault syndrome OMIM:606075	*TWNK*	NM_021830.5	c.1172G>A p.Arg391His	LP	c.1217G>A p.Arg406Gln	LP	Yes
	1	Perrault syndrome OMIM:606075	*TWNK*	NM_021830.5	c.1172G>A p.Arg391His	LP	c.1844G>C p.Gly615Ala	LP	Yes

P: pathogenic; LP: likely pathogenic; VUS: variant of uncertain significance; N/A: not applicable.

**Table 4:** Ethnicity of AN cases with a genetic diagnosis—organised according to the gene in which the diagnosis was made.

Gene	Ethnicity	No. of Cases	References—Author (Year)
*JAM3*	North African and Middle Eastern	1	Abdallah Moady (2023) [22]
*OTOF*	South American	2	Batissoco (2022) [4]
	Southern and Eastern European (Italian)	1	Forli (2023) [26]
	Northeast Asian (Japanese)	35	Iwasa (2022) [30]
	Northeast Asian (Chinese)	44	Jiang (2022) [32], Liu (2022) [37], Qiu (2019) [38], Wang (2018) [41], Wu (2023) [45], Zhai (2020) [47], Zhang (2016) [48], Zhu (2021) [49]
	Northeast Asian (Korean)	11	Kim (2018) [33]
	Northeast Asian (Taiwanese)	18	Lin (2022) [36]
*XKR8*	Northeast Asian (Chinese)	4	Chen (2023) [23]
*SH3TC2*	Southern and Central Asian (Indian)	1	Chhajed (2022) [24]
*FLG*	Southern and Central Asian (Indian)	1	Chhajed (2022) [24]
*PJVK*	Southern and Eastern European (Italian)	1	Domínguez-Ruiz (2022) [25]
*OPA1*	Northeast Asian (Japanese)	1	Ideura (2019) [29]
	Northeast Asian (Korean)	2	Kim (2023) [34]
	Northeast Asian (Taiwanese)	1	Lin (2022) [36]
*TMEM43*	Northeast Asian (Korean, Chinese)	15	Jang (2021) [31], Kim (2023) [34]
*PMP22*	Northeast Asian (Korean)	1	Kim (2023) [34]
*ATP1A3*	Northeast Asian (Korean)	2	Kim (2023) [34]
	Northeast Asian (Chinese)	5	Wang (2021) [43], Wu (2023) [45]
*MYO3A*	Northeast Asian (Chinese)	1	Liu (2022) [37]
*PLA2G6*	Southeast Asian	2	Li (2020) [35]
*WFS1*	Northeast Asian (Chinese)	3	Lin (2022) [36], Sun (2022) [39]
*TIMM8A*	Northeast Asian (Chinese)	3	Sun (2022) [39], Wu (2023) [45]
*MITF*	Northeast Asian (Chinese)	1	Sun (2022) [39]
*LARS2*	Northeast Asian (Chinese)	1	Sun (2022) [39]
*AIFM1*	Northeast Asian (Chinese)	23	Wang (2020) [42], Wu (2023) [45], Zanin (2020) [46]
*MT-CO1*	Northeast Asian (Chinese)	1	Wu (2023) [45]
*ACTG1*	Northeast Asian (Chinese)	1	Wu (2023) [45]
*TWIST1*	Northeast Asian (Chinese)	1	Wu (2023) [45]
*TWNK*	Northeast Asian (Chinese)	2	Wu (2023) [45]
*DIAPH1*	Northeast Asian (Chinese)	4	Wu (2020) [44]

**Table 5:** Characteristics of AN hearing loss amongst cases with a genetic diagnosis: Information on severity, CNH, laterality, and age of onset is noted here when reported by the authors of the studies.

Gene	No. of Cases	Hearing Loss Severity (PTA/Behavioural Audiometry)	Cochlear Nerve Hypoplasia (CNH)	Hearing Loss Laterality	Age of AN Onset	Author (Year)
*JAM3*	1	Profound	Not reported	Bilateral	<3 months	Abdallah Moady (2023) [22]
*OTOF*	107	Most have severe-profound HL	Not reported	Bilateral	Most pre-lingual	Batissoco (2022) [4], Hosoya (2018) [28], Iwasa (2022) [30], Jiang (2022) [32], Kim (2018) [33], Liu (2022) [37], Lin (2022) [36], Qiu (2019) [38], Sun (2022) [39], Tang (2015) [40], Wang (2018) [41], Wu (2023) [45], Zhai (2020) [47], Zhang (2016) [48]
*OTOF* (Temperature-sensitive AN)	9	Febrile: mild–severe Afebrile: normal–moderately severe	Not reported	Bilateral	<3 years (4 cases) 6 years (1 case) ‘Childhood onset’ (4 cases)	Forli (2023) [26], Zhang (2016) [48], Zhu (2021) [49]
*XKR8*	4	Not reported	Not reported	Bilateral	20–40 years	Chen (2023) [23]
*SH3TC2/FLG*	1	Not reported	Not reported	Bilateral	3 years	Chhajed (2022) [24]
*PJVK*	1	Profound	Not reported	Bilateral	<2 years	Domínguez-Ruiz (2022) [25]
*NFASC*	2	Not reported	Not reported	Bilateral	<10 years (1 case) <8 months (1 case)	Harper (2020) [27]
*OPA1*	4	Mild–severe (most were moderate)	Bilateral CNH (1 case)	Bilateral	Post-lingual (2 cases)	Ideura (2019) [29], Kim (2023) [34], Lin (2022) [36]
*TMEM43*	15	Mild–moderate (1 case) Elevated PTA threshold (14 pts)	Not reported	Not reported	Most around 25 years	Jang (2021) [31], Kim (2023) [34]
*PMP22*	1	Mild–moderate	Not reported	Not reported	Post-lingual	Kim (2023) [34]
*ATP1A3*	7	Mild–severe	Bilateral CNH (1 case)	Bilateral	Post-lingual (4–9 years)	Kim (2023) [34], Wang (2021) [43], Wu (2023) [45]
*MYO3A*	1	Profound	Not reported	Not reported	<6 years	Liu (2022) [37]
*PLA2G6*	2	Not reported	Not reported	Bilateral	<2 years	Li (2020) [35]
*WFS1*	3	Profound	Not reported	Bilateral	<3 months (1 case) 15–53 months (2 cases)	Lin (2022) [36] Sun (2022) [39]
*TIMM8A*	3	Not reported	Not reported	Bilateral	15–53 months (1 case) 2 years (1 case) 0.17 years (1 case)	Sun (2022) [39] Wu (2023) [45]
*MITF*	1	Not reported	Not reported	Not reported	15–53 months	Sun (2022) [39]
*LARS2*	1	Not reported	Not reported	Not reported	15–53 months	Sun (2022) [39]
*AIFM1*	23	Mild–severe	Bilateral CNH	Bilateral	6–20 years	Wang (2020) [42], Wu (2023) [45], Zanin (2020) [46]
*MT-CO1*	1	Profound	Not reported	Bilateral	0.5 years	Wu (2023) [45]
*ACTG1*	1	Severe	Not reported	Bilateral	1 year	Wu (2023) [45]
*TWIST1*	1	Profound	Not reported	Bilateral	0 years	Wu (2023) [45]
*TWNK*	2	Profound	Not reported	Bilateral	0–5.5 years	Wu (2023) [45]
*DIAPH1*	4	Moderate–severe	Not reported	Bilateral	20–35 years	Wu (2020) [44]

## Data Availability

The original contributions presented in this study are included in the article/Supplementary Material. Further inquiries can be directed to the corresponding author(s).

## Supplementary Materials

The following supporting information can be downloaded at https://www.mdpi.com/article/10.3390/jcm15114260/s1, Table S1: Search strategy; Table S2: Critical appraisal for case reports using the JBI checklist; Table S3: Critical appraisal for case series using the JBI checklist; Table S4: Critical appraisal for case-control studies using the JBI checklist; Table S5: Critical appraisal for cohort studies using the JBI checklist; Table S6: Critical appraisal for cross-sectional studies using the JBI checklist; Table S7: Overview of genetic aetiology and postulated site of lesion along the hearing pathway. File S1: PRISMA 2020 checklist. References [67,68,69,70,71,72,73,74,75,76,77] are cited in the Supplementary Materials only.

## Abbreviations

The following abbreviations are used in this manuscript:

AN	Auditory neuropathy
OAEs	Otoacoustic emissions
ABR	Auditory brainstem response
CM	Cochlear microphonic
PTA	Pure tone audiometry
IHC	Inner hair cell
ACMG	American College of Medical Genetics and Genomics
VUS	Variant of uncertain significance
LP	Likely pathogenic
P	Pathogenic
CNH	Cochlear nerve hypoplasia
NGS	Next generation sequencing
AR	Autosomal recessive
OMIM	Online Mendelian Inheritance in Man
