# Supplementary material for: The Genetic Causes of Auditory Neuropathy: A Systematic Review
Source: J Clin Med. 2026 May 31;15(11):4260. doi: 10.3390/jcm15114260 (PMC13258488; doi:10.3390/jcm15114260)
Supplement: Supplementary file 1 [file jcm-15-04260-s001.zip › jcm-4307919 - Supplementary material.pdf]

Journal of Clinical Medicine

SUPPLEMENTARY MATERIAL

Table S1. Search Strategy

**Ovid MEDLINE(R) was searched via OvidSP. The database coverage was from 1946 to present and the database was searched on 29 January 2024.**<br>1 exp *Auditory Diseases, Central/di, et, ge [Diagnosis, Etiology, Genetics]<br>2 (auditory-neuropath* or ANSD or auditory-dyssynchrony).tw,kf.<br>3 exp *Vestibulocochlear Nerve Diseases/di, et, ge [Diagnosis, Etiology, Genetics]<br>4 (gene* or autosomal or syndrom* or X-linked or Xlinked or mutation or genom* or genotyp* or mitochondria* or variant* or pathogenic or recessive or dominant or DNA).tw,kf,hw.<br>5 (1 or 2 or 3) and 4<br>6 limit 5 to (comment or editorial or guideline or letter or practice guideline or preprint)<br>7 5 not 6
**Embase Classic+Embase was searched via OvidSP. The database coverage was from 1947 to present and the database was searched on 29 January 2024.**<br>1 *perception deafness/ or exp *central hearing loss/<br>2 (auditory-neuropath* or ANSD or auditory-dyssynchrony).tw,kf,dq.<br>3 *vestibulocochlear nerve disease/<br>4 (gene* or autosomal or syndrom* or X-linked or Xlinked or mutation or genom* or genotyp* or mitochondria* or variant* or pathogenic or recessive or dominant or DNA).tw,kf,hw,dq.<br>5 (1 or 2 or 3) and 4<br>6 limit 5 to (conference abstract or conference paper or "conference review" or editorial or letter or "preprint (unpublished, non-peer reviewed)")<br>7 5 not 6
**PubMed was searched on 29 January 2024 with no date restrictions, database coverage to the present.**<br>#1 Title/Abstract<br>"auditory-neuropath*" OR "ANSD" OR "auditory-dyssynchrony" OR "perception-deafness" OR "central-auditory-disease*" OR "vestibulocochlear-nerve-disease*"<br>#2 Title/Abstract<br>"gene*" OR "autosomal" OR "syndrom*" OR "X-linked" OR "Xlinked" OR "mutation" OR "genom*" OR "genotype*" OR "mitochondria*" OR "variant*" OR "pathogenic" OR "recessive" OR "dominant" OR "DNA"<br>#3 All fields<br>NOTNLM OR publisher[sb] OR inprocess[sb] OR pubmednotmedline[sb] OR indatareview[sb] OR pubstatusaheadofprint<br>#4 #1 AND #2 AND #3<br>excluded books and documents OR comment OR editorial OR guideline OR letter OR practice guideline OR preprint

Table S2. Critical appraisal for case reports using the JBI checklist

Author (year)	Q1	Q2	Q3	Q4	Q5	Q6	Q7
Chhajed (2022)[1]	Yes	Yes	Yes	Yes	N/A	N/A	Yes
Li (2020)[2]	Yes	Yes	Yes	Yes	N/A	N/A	Yes
Jiang (2022)[3]	Yes	Yes	Yes	Yes	N/A	N/A	Yes
Harper (2020)[4]	Yes	Yes	Yes	Yes	Yes	Yes	Yes
Forli (2023)[5]	Yes	Yes	Yes	Yes	Yes	Yes	Yes
Abdallah Moady (2023)[6]	Yes	Yes	Yes	Yes	N/A	N/A	Yes

Q1: Were patient's demographic characteristics clearly described?
Q2: Was the patient's history clearly described and presented as a timeline?
Q3: Was the current clinical condition of the patient on presentation clearly described?
Q4: Were diagnostic tests or assessment methods and the results clearly described?
Q5: Was the intervention(s) or treatment procedure(s) clearly described?
Q6: Was the post-intervention clinical condition clearly described?
Q7: Does the case report provide takeaway lessons?

Table S3. Critical appraisal for case series using the JBI checklist

Author (year)	Q1	Q2	Q3	Q4	Q5	Q6	Q7	Q8	Q9	Q10
Zhai (2020)[7]	Yes	Yes	Yes	No	No	Yes	Yes	No	Yes	N/A
Qiu (2019)[8]	Yes	Yes	Yes	No	No	Yes	Yes	Yes	Yes	N/A
Zhu (2021)[9]	Yes	Yes	Yes	No	No	Yes	Yes	N/A	No	Yes
Zhang (2016)[10]	Yes	Yes	Yes	Unclear	Unclear	Yes	Yes	No	Yes	Yes

Wu (2020)[11]	Yes	Yes	Yes	No	No	No	Yes	Yes	Yes	N/A
Wang (2018)[12]	Yes	No	No	No	No	No	Yes	N/A	No	N/A
Wang (2021)[13]	Yes	Yes	Yes	No	Unclear	Yes	Yes	Yes	Yes	N/A
Tang (2015)[14]	Yes	No	Unclear	No	Unclear	No	Yes	N/A	No	N/A
Sun (2022)[15]	Yes	Yes	Yes	No	Unclear	No	Yes	N/A	Yes	N/A
Kim (2018)[16]	Yes	Yes	Yes	No	Unclear	No	Yes	Yes	Yes	Yes
Jang (2021)[17]	Yes	Yes	Yes	No	No	No	Yes	Yes	No	Yes
Hosoya (2018)[18]	Yes	Unclear	Unclear	Unclear	No	Yes	No	Yes	No	Yes
Chen (2023)[19]	Yes	Yes	Yes	No	No	Yes	Yes	N/A	Yes	Yes

Q1: Were there clear criteria for inclusion in the case series?
Q2: Was the condition measured in a standard, reliable way for all participants included in the case series?
Q3: Were valid methods used for identification of the condition for all participants included in the case series?
Q4: Did the case series have consecutive inclusion of participants?
Q5: Did the case series have complete inclusion of participants?
Q6: Was there clear reporting of the demographics of the participants in the study?
Q7: Was there clear reporting of clinical information of the participants?
Q8: Were the outcomes or follow up results of cases clearly reported?
Q9: Was there clear reporting of the presenting site(s)/clinic(s) demographic information?
Q10: Was statistical analysis appropriate?

Table S4. Critical appraisal for case-control studies using the JBI checklist

Author (year)	Q1	Q2	Q3	Q4	Q5	Q6	Q7	Q8	Q9
Zanin (2020) [20]	Yes	Yes	Unclear	Yes	Unclear	No	Yes	Yes	Yes

Q1: Were the groups comparable other than the presence of disease in cases or the absence of disease in controls?
Q2: Were cases and controls matched appropriately?
Q3: Were the same criteria used for identification of cases and controls?
Q4: Was exposure measured in a standard, valid and reliable way?
Q5: Was exposure measured in the same way for cases and controls?
Q6: Were confounding factors identified?
Q7: Were outcomes assessed in a standard, valid and reliable way for cases and controls?
Q8: Was the exposure period of interest long enough to be meaningful?
Q9: Was appropriate statistical analysis used?

Table S5. Critical appraisal for cohort studies using the JBI checklist

Author (year)	Q1	Q2	Q3	Q4	Q5	Q6	Q7
Wang (2020)[21]	Yes	No	Yes	Yes	No	No	Yes
Kim (2023)[22]	Yes	Yes	Yes	Yes	Yes	N/A	Yes
Iwasa (2022)[23]	Yes	N/A	Yes	Yes	Yes	N/A	Yes
Wu (2023)[24]	Yes	Yes	Yes	Yes	No	No	Yes
Lin (2022)[25]	Yes	Yes	Yes	Yes	Yes	N/A	Yes

Q1: Was the exposure measured in a valid and reliable way?
Q2: Were the participants free of the outcome at the start of the study (or at the moment of exposure)?
Q3: Were the outcomes measured in a valid and reliable way?
Q4: Was the follow up time reported and sufficient to be long enough for outcomes to occur?
Q5: Was follow up complete, and if not, were the reasons to loss to follow up described and explored?
Q6: Were strategies to address incomplete follow up utilized?
Q7: Was appropriate statistical analysis used?

Table S6. Critical appraisal for cross-sectional studies using the JBI checklist

Author (year)	Q1	Q2	Q3	Q4	Q5	Q6
Liu (2022)[26]	Yes	Yes	Yes	Yes	Yes	Yes
Ideura (2019)[27]	Yes	Yes	Yes	Yes	Yes	Yes
Batissoco (2022)[28]	Yes	Yes	Yes	Yes	Yes	Yes

Q1: Were the criteria for inclusion clearly defined?
Q2: Were the study subjects and the setting described in detail?
Q3: Was the exposure measured in a valid and reliable way?
Q4: Were objective, standard criteria used for measurement of the condition?
Q5: Were the outcomes measured in a valid and reliable way?
Q6: Was appropriate statistical analysis used?

https://doi.org/10.3390/jcm15114260

Table S7. Overview of genetic aetiology and postulated site of lesion along the hearing pathway

Locus / Mechanism	Aetiology	References – Author (Year)
Cochlea inner hair cells	*DIAPH1*	Lynch (1997)[29]
Cochlear glia-like supporting cells	*TMEM43*	Jang (2021)[17]
Ribbon synapse	*OTOF*	Roux (2006)[30]<br>Pangrsic (2010)[31]
Terminal dendrites of auditory nerve fibres	*OPA1*	Huang (2009)[32]<br>Santarelli (2015)[33]
Predominantly expressed in spiral ganglion cells<br>(and weakly expressed in the bottom of inner hair cells)	*XKR8*	Chen (2023)[19]
ATP1A3 expressed in spiral ganglion neurons (type I afferent terminals, spiral ganglion somata)	*ATP1A3*	McLean (2009)[34]<br>Kim (2023)[22]
Degeneration of spiral ganglion cells (associated with Mohr-Tranebjaerg syndrome)	*TIMM8A*	Merchant (2001)[35]<br>Bahmad (2007)[36]
Axonal degeneration of the auditory nerve	*AIFM1*	Wang (2020)[21]<br>Zanin (2020)[20]
Myelin (associated with Charcot-Marie Tooth disease type 1A – a hereditary demyelinating nerve disorder)	*PMP22*	Rance (2012)[37]
Hypothesised that *JAM3* mutations cause AN through endothelial microvasculature disruption of cranial nerve VIII or impairment of nerve conduction from the synapse to brainstem	*JAM3*	Abdallah Moady (2023)[6]
Postulated to affect stereocilia of IHCs and/or mediate autophagic degradation of peroxisomes to protect against oxidative stress in noise over-exposure	*PJVK*	Kazmierczak (2017)[38]
Hypothesised that the function of *MITF* is key in melanocyte survival and differentiation. Absence of cochlear melanocytes in the cochlea may cause hearing loss (associated with Waardenburg Syndrome 2A)	*MITF*	Tachibana (1992)[39]<br>Tachibana (1996)[40]
Hypothesised that *LARS2* mutations lead to inadequate mitochondrial function in inner ear. Perturbations in mitochondrial protein translation can lead to hearing loss through tissue-specific apoptosis.	*LARS2*	Pierce (2013)[41]<br>Raimundo (2012)[42]

https://doi.org/10.3390/jcm15114260

1. Chhajed, M.; Gunasekaran, P.K.; Bhanudeep, S.; Saini, L. Charcot-Marie-Tooth Disease Type 4C and Autosomal Dominant Heterozygous Ichthyosis Vulgaris, with Bilateral Hearing Loss: A Novel Association with Review of Literature. *J Pediatr Genet* **2022**, *13*, 110–115, doi:10.1055/s-0042-1759780.
2. Li, L.; Fong, C.Y.; Tay, C.G.; Tae, S.K.; Suzuki, H.; Kosaki, K.; Thong, M.K. Infantile neuroaxonal dystrophy in a pair of Malaysian siblings with progressive cerebellar atrophy: Description of an expanded phenotype with novel PLA2G6 variants. *J Clin Neurosci* **2020**, *71*, 289–292, doi:10.1016/j.jocn.2019.08.111.
3. Jiang, L.; Xu, H.; Liu, D.; Zhang, S.; Xu, Y. Case report: Clinical and genetic analysis of a family with nonsyndromic auditory neuropathy. *Front Pediatr* **2022**, *10*, Article 1005335, doi:10.3389/fped.2022.1005335.
4. Harper, J.L.; Wilson, T.E.; Mitchell, R.M. Case report of two children with auditory neuropathy spectrum disorder related to a neurofascin (NFASC) gene variant. *Int J Pediatr Otorhinolaryngol* **2020**, *131*, Article 109863, doi:10.1016/j.ijporl.2020.109863.
5. Forli, F.; Capobianco, S.; Berrettini, S.; Bruschini, L.; Romano, S.; Fogli, A.; Bertini, V.; Lazzerini, F. Temperature-Sensitive Auditory Neuropathy: Report of a Novel Variant of OTOF Gene and Review of Current Literature. *Medicina* **2023**, *59*, Article 352, doi:10.3390/medicina59020352.
6. Abdallah Moady, T.; Odeh, M.; Fedida, A.; Segal, Z.; Gruber, M.; Goldfeld, M.; Kalfon, L.; Falik-Zaccai, T.C. Case report: Novel insights into hemorrhagic destruction of the brain, subependymal calcification, and cataracts disease. *Front Pediatr* **2023**, *11*, Article 1178280, doi:10.3389/fped.2023.1178280.
7. Zhai, R.; Feng, H.; Li, Q.; Lu, W.; Liu, D.; Tian, Y.; Liu, H.; Li, R.; Zuo, B.; Tang, W.; et al. Auditory Neuropathy Spectrum Disorder (ANSD)-Clinical Characteristics and Pathogenic Variant Analysis of Three Nonsyndromic Deafness Families. *BioMed Res Int* **2020**, *2020*, Article 8843539, doi:10.1155/2020/8843539.
8. Qiu, Y.; Chen, S.; Xie, L.; Xu, K.; Lin, Y.; Bai, X.; Zhang, H.-M.; Liu, X.-Z.; Jin, Y.; Sun, Y.; et al. Auditory Neuropathy Spectrum Disorder due to Two Novel Compound Heterozygous OTOF Mutations in Two Chinese Families. *Neural Plast* **2019**, *2019*, Article 9765276, doi:10.1155/2019/9765276.
9. Zhu, Y.M.; Li, Q.; Gao, X.; Li, Y.F.; Liu, Y.L.; Dai, P.; Li, X.P. Familial Temperature-Sensitive Auditory Neuropathy: Distinctive Clinical Courses Caused by Variants of the OTOF Gene. *Front Cell Dev Biol* **2021**, *9*, Article 732930, doi:10.3389/fcell.2021.732930.
10. Zhang, Q.J.; Han, B.; Lan, L.; Zong, L.; Shi, W.; Wang, H.Y.; Xie, L.Y.; Wang, H.; Zhao, C.; Zhang, C.; et al. High frequency of OTOF mutations in Chinese infants with congenital auditory neuropathy spectrum disorder. *Clin Genet* **2016**, *90*, 238–246, doi:10.1111/cge.12744.
11. Wu, K.; Wang, H.; Guan, J.; Lan, L.; Zhao, C.; Zhang, M.; Wang, D.; Wang, Q. A novel variant in diaphanous homolog 1 (DIAPH1) as the cause of auditory neuropathy in a Chinese family. *Int J Pediatr Otorhinolaryngol* **2020**, *133*, Article 109947, doi:10.1016/j.ijporl.2020.109947.
12. Wang, Y.; Lu, Y.; Cheng, J.; Zhang, L.; Han, D.; Yuan, H. Novel OTOF gene mutations identified using a massively parallel DNA sequencing technique in DFNB9 deafness. *Acta Otolaryngol* **2018**, *138*, 865–870, doi:10.1080/00016489.2018.1476777.

13. Wang, W.; Li, J.; Lan, L.; Xie, L.; Xiong, F.; Guan, J.; Wang, H.; Wang, Q. Auditory Neuropathy as the Initial Phenotype for Patients With ATP1A3 c.2452 G > A: Genotype-Phenotype Study and CI Management. *Front Cell Dev Biol* **2021**, *9*, Article 749484, doi:10.3389/fcell.2021.749484.
14. Tang, H.Y.; Fang, P.; Lin, J.W.; Darilek, S.; Osborne, B.T.; Haymond, J.A.; Manolidis, S.; Roa, B.B.; Oghalai, J.S.; Alford, R.L. DNA sequence analysis and genotype-phenotype assessment in 71 patients with syndromic hearing loss or auditory neuropathy. *BMJ Open* **2015**, *5*, Article e007506, doi:10.1136/bmjopen-2014-007506.
15. Sun, L.; Lin, Z.; Zhang, J.; Shen, J.; Wang, X.; Yang, J. Genetic etiological analysis of auditory neuropathy spectrum disorder by next-generation sequencing. *Front Neurol* **2022**, *13*, Article 1026695, doi:10.3389/fneur.2022.1026695.
16. Kim, B.J.; Jang, J.H.; Han, J.H.; Park, H.R.; Oh, D.Y.; Lee, S.; Kim, M.Y.; Kim, A.R.; Lee, C.; Kim, N.K.D.; et al. Mutational and phenotypic spectrum of OTOF-related auditory neuropathy in Koreans: eliciting reciprocal interaction between bench and clinics. *J Transl Med* **2018**, *16*, Article 330, doi:10.1186/s12967-018-1708-z.
17. Jang, M.W.; Oh, D.Y.; Yi, E.; Liu, X.; Ling, J.; Kim, N.; Sharma, K.; Kim, T.Y.; Lee, S.; Kim, A.R.; et al. A nonsense TMEM43 variant leads to disruption of connexin-linked function and autosomal dominant auditory neuropathy spectrum disorder. *Proc Natl Acad Sci U S A* **2021**, *118*, Article e2019681118, doi:10.1073/pnas.2019681118.
18. Hosoya, M.; Minami, S.B.; Enomoto, C.; Matsunaga, T.; Kaga, K. Elongated EABR wave latencies observed in patients with auditory neuropathy caused by OTOF mutation. *Laryngoscope Investig Otolaryngol* **2018**, *3*, 388–393, doi:10.1002/lio2.210.
19. Chen, K.; Li, C.; Dong, C.; Cen, X.; Wang, Y.; Liang, Y.; Zhu, Y.; Fang, S.; Jiang, H. A dominant variant in apoptosis-related gene XKR8 is relevant to hereditary auditory neuropathy. *J Transl Med* **2023**, *21*, Article 279, doi:10.1186/s12967-023-04139-x.
20. Zanin, J.; Dhollander, T.; Rance, G.; Yu, L.; Lan, L.; Wang, H.; Lou, X.; Connelly, A.; Nayagam, B.; Wang, Q. Fiber-Specific Changes in White Matter Microstructure in Individuals With X-Linked Auditory Neuropathy. *Ear Hear* **2020**, *41*, 1703–1714, doi:10.1097/AUD.0000000000000890.
21. Wang, H.; Bing, D.; Li, J.; Xie, L.; Xiong, F.; Lan, L.; Wang, D.; Guan, J.; Wang, Q. High Frequency of AIFM1 Variants and Phenotype Progression of Auditory Neuropathy in a Chinese Population. *Neural Plast* **2020**, *2020*, Article 5625768, doi:10.1155/2020/5625768.
22. Kim, Y.; Han, J.J.; Oh, J.; Han, J.H.; Kim, M.Y.; Jung, J.; Choi, J.Y.; Choi, B.Y. Audiogram Configuration, Molecular Etiology, and Outcome of Cochlear Implantation in Postlingual Auditory Neuropathy Spectrum Disorder. *Otol Neurotol* **2023**, *44*, e471–e478, doi:10.1097/MAO.0000000000003915.
23. Iwasa, Y.-I.; Nishio, S.-Y.; Yoshimura, H.; Sugaya, A.; Kataoka, Y.; Maeda, Y.; Kanda, Y.; Nagai, K.; Naito, Y.; Yamazaki, H.; et al. Detailed clinical features and genotype-phenotype correlation in an OTOF-related hearing loss cohort in Japan. *Hum Genet* **2022**, *141*, 865–875, doi:10.1007/s00439-021-02351-7.
24. Wu, J.; Chen, J.; Ding, Z.; Fan, J.; Wang, Q.; Dai, P.; Han, D. Outcomes of cochlear implantation in 75 patients with auditory neuropathy. *Front Neurosci* **2023**, *17*, Article 1281884, doi:10.3389/fnins.2023.1281884.

25. Lin, P.H.; Wu, H.P.; Wu, C.M.; Chiang, Y.T.; Hsu, J.S.; Tsai, C.Y.; Wang, H.; Tseng, L.H.; Chen, P.Y.; Yang, T.H.; et al. Cochlear Implantation Outcomes in Patients with Auditory Neuropathy Spectrum Disorder of Genetic and Non-Genetic Etiologies: A Multicenter Study. *Biomedicines* **2022**, *10*, Article 1523, doi:10.3390/biomedicines10071523.
26. Liu, Y.; Tan, M.; Cai, L.; Lv, L.; Chen, Q.; Chen, W.; Yang, H.; Xu, Y. Genetic profiles of non-syndromic severe-profound hearing loss in Chinese Hans by whole-exome sequencing. *Gene* **2022**, *819*, Article 146258, doi:10.1016/j.gene.2022.146258.
27. Ideura, M.; Nishio, S.-Y.; Moteki, H.; Takumi, Y.; Miyagawa, M.; Sato, T.; Kobayashi, Y.; Ohyama, K.; Oda, K.; Matsui, T.; et al. Comprehensive analysis of syndromic hearing loss patients in Japan. *Sci Rep* **2019**, *9*, Article 11976, doi:10.1038/s41598-019-47141-4.
28. Batissoco, A.C.; Pedroso-Campos, V.; Pardono, E.; Sampaio-Silva, J.; Sonoda, C.Y.; Vieira-Silva, G.A.; da Silva de Oliveira Longati, E.U.; Mariano, D.; Hoshino, A.C.H.; Tsuji, R.K.; et al. Molecular and genetic characterization of a large Brazilian cohort presenting hearing loss. *Hum Genet* **2022**, *141*, 519–538, doi:10.1007/s00439-021-02372-2.
29. Lynch, E.; Lee, M.; Morrow, J.; Welcsh, P.; León, P.; King, M.-C. Nonsyndromic deafness DFNA1 associated with mutation of the human homolog HDIA1 of the Drosophila diaphanous gene. *Science* **1997**, *278*, 1315–1318, doi:10.1126/science.278.5341.1315.
30. Roux, I.; Safieddine, S.; Nouvian, R.; Grati, M.h.; Simmler, M.-C.; Bahloul, A.; Perfettini, I.; Le Gall, M.; Rostaing, P.; Hamard, G.; et al. Otoferlin, Defective in a Human Deafness Form, Is Essential for Exocytosis at the Auditory Ribbon Synapse. *Cell* **2006**, *127*, 277–289, doi:10.1016/j.cell.2006.08.040.
31. Pangršič, T.; Lasarow, L.; Reuter, K.; Takago, H.; Schwander, M.; Riedel, D.; Frank, T.; Tarantino, L.M.; Bailey, J.S.; Strenzke, N.; et al. Hearing requires otoferlin-dependent efficient replenishment of synaptic vesicles in hair cells. *Nat Neurosci* **2010**, *13*, 869–876, doi:10.1038/nn.2578.
32. Huang, T.; Santarelli, R.; Starr, A. Mutation of OPA1 gene causes deafness by affecting function of auditory nerve terminals. *Brain Res* **2009**, *1300*, 97–104, doi:10.1016/j.brainres.2009.08.083.
33. Santarelli, R.; Rossi, R.; Scimemi, P.; Cama, E.; Valentino, M.L.; La Morgia, C.; Caporali, L.; Liguori, R.; Magnavita, V.; Monteleone, A.; et al. OPA1-related auditory neuropathy: site of lesion and outcome of cochlear implantation. *Brain* **2015**, *138*, 563–576, doi:10.1093/brain/awu378.
34. McLean, W.J.; Smith, K.A.; Glowatzki, E.; Pyott, S.J. Distribution of the Na,K-ATPase alpha subunit in the rat spiral ganglion and organ of corti. *J Assoc Res Otolaryngol* **2009**, *10*, 37–49, doi:10.1007/s10162-008-0152-9.
35. Merchant, S.N.; McKenna, M.J.; Nadol, J.B., Jr.; Kristiansen, A.G.; Tropitzsch, A.; Lindal, S.; Tranebjaeizrg, L. Temporal bone histopathologic and genetic studies in Mohr-Tranebjaerg syndrome (DFN-1). *Otol Neurotol* **2001**, *22*, 506–511, doi:10.1097/00129492-200107000-00017.
36. Bahmad, F., Jr.; Merchant, S.N.; Nadol, J.B., Jr.; Tranebjaerg, L. Otopathology in Mohr-Tranebjaerg syndrome. *Laryngoscope* **2007**, *117*, 1202–1208, doi:10.1097/MLG.0b013e3180581944.
37. Rance, G.; Ryan, M.M.; Bayliss, K.; Gill, K.; O'Sullivan, C.; Whitechurch, M. Auditory function in children with Charcot-Marie-Tooth disease. *Brain* **2012**, *135*, 1412–1422, doi:10.1093/brain/aws085.

38. Kazmierczak, M.; Kazmierczak, P.; Peng, A.W.; Harris, S.L.; Shah, P.; Puel, J.L.; Lenoir, M.; Franco, S.J.; Schwander, M. Pejvakin, a Candidate Stereociliary Rootlet Protein, Regulates Hair Cell Function in a Cell-Autonomous Manner. *J Neurosci* **2017**, *37*, 3447–3464, doi:10.1523/jneurosci.2711-16.2017.
39. Tachibana, M.; Hara, Y.; Vyas, D.; Hodgkinson, C.; Fex, J.; Grundfast, K.; Arnheiter, H. Cochlear disorder associated with melanocyte anomaly in mice with a transgenic insertional mutation. *Mol Cell Neurosci* **1992**, *3*, 433–445, doi:10.1016/1044-7431(92)90055-7.
40. Tachibana, M.; Takeda, K.; Nobukuni, Y.; Urabe, K.; Long, J.E.; Meyers, K.A.; Aaronson, S.A.; Miki, T. Ectopic expression of MITF, a gene for Waardenburg syndrome type 2, converts fibroblasts to cells with melanocyte characteristics. *Nat Genet* **1996**, *14*, 50–54, doi:10.1038/ng0996-50.
41. Pierce, S.B.; Gersak, K.; Michaelson-Cohen, R.; Walsh, T.; Lee, M.K.; Malach, D.; Klevit, R.E.; King, M.C.; Levy-Lahad, E. Mutations in LARS2, encoding mitochondrial leucyl-tRNA synthetase, lead to premature ovarian failure and hearing loss in Perrault syndrome. *Am J Hum Genet* **2013**, *92*, 614–620, doi:10.1016/j.ajhg.2013.03.007.
42. Raimundo, N.; Song, L.; Shutt, Timothy E.; McKay, Sharen E.; Cotney, J.; Guan, M.-X.; Gilliland, Thomas C.; Hohuan, D.; Santos-Sacchi, J.; Shadel, Gerald S. Mitochondrial Stress Engages E2F1 Apoptotic Signaling to Cause Deafness. *Cell* **2012**, *148*, 716–726, doi:10.1016/j.cell.2011.12.027.

https://doi.org/10.3390/jcm15114260
